# Anxiety, depression and behavioral problems among adolescents with recurrent headache: the Young-HUNT study

**DOI:** 10.1186/1129-2377-15-38

**Published:** 2014-06-13

**Authors:** Brit A Blaauw, Grete Dyb, Knut Hagen, Turid L Holmen, Mattias Linde, Tore Wentzel-Larsen, John-Anker Zwart

**Affiliations:** 1 Department of Neurology, Vestfold Hospital, Tønsberg, Norway; 2 Faculty of medicine, Institute of Clinical Medicine, University of Oslo, Oslo, Norway; 3 Norwegian Centre for Violence and Traumatic Stress Studies, Oslo, Norway; 4 Department of Neuroscience, Faculty of Medicine, Norwegian University of Science and Technology, Trondheim, Norway; 5 Norwegian National Headache Centre, Section of Neurology, St. Olavs Hospital, Trondheim, Norway; 6 HUNT research Centre, Department of Public Health and General Practice, Faculty of Medicine, Norwegian University of Science and Technology, Trondheim, Norway; 7 Center for Child and Adolescent Mental Health, Eastern and Southern Norway, Oslo, Norway; 8 Department of Neurology and FORMI, Oslo University Hospital, Ullevål, Oslo, Norway

**Keywords:** Recurrent headache, Migraine, Tension-type headache, Anxiety, Depression, Behavioral problems, Conduct difficulties, Attention difficulties, Adolescents

## Abstract

**Background:**

It is well documented that both anxiety and depression are associated with headache, but there is limited knowledge regarding the relation between recurrent primary headaches and symptoms of anxiety and depression as well as behavioral problems among adolescents. Assessment of co-morbid disorders is important in order to improve the management of adolescents with recurrent headaches. Thus the main purpose of the present study was to assess the relationship of recurrent headache with anxiety and depressive symptoms and behavioral problems in a large population based cross-sectional survey among adolescents in Norway.

**Methods:**

A cross-sectional, population-based study was conducted in Norway from 1995 to 1997 (Young-HUNT1). In Young-HUNT1, 4872 adolescents aged 12 to 17 years were interviewed about their headache complaints and completed a comprehensive questionnaire that included assessment of symptoms of anxiety and depression and behavioral problems, i.e. conduct and attention difficulties.

**Results:**

In adjusted multivariate analyses among adolescents aged 12–14 years, recurrent headache was associated with symptoms of anxiety and depression (OR: 2.05, 95% CI: 1.61-2.61, *p* < 0.001), but not with behavioral problems. A significant association with anxiety and depressive symptoms was evident for all headache categories; i.e. migraine, tension-type headache and non-classifiable headache. Among adolescents aged 15–17 years there was a significant association between recurrent headache and symptoms of anxiety and depression (OR: 1.64, 95% CI: 1.39-1.93, *p* < 0,001) and attention difficulties (OR: 1.25, 95% CI: 1.09-1.44, *p* =0.001). For migraine there was a significant association with both anxiety and depressive symptoms and attention difficulties, while tension-type headache was significantly associated only with symptoms of anxiety and depression. Non-classifiable headache was associated with attention difficulties and conduct difficulties, but not with anxiety and depressive symptoms. Headache frequency was significantly associated with increasing symptoms scores for anxiety and depressive symptoms as well as attention difficulties, evident for both age groups.

**Conclusions:**

The results from the present study indicate that both anxiety and depressive symptoms and behavioral problems are associated with recurrent headache, and should accordingly be considered a part of the clinical assessment of children and adolescents with headache. Identification of these associated factors and addressing them in interventions may improve headache management.

## Background

Recurrent headaches are common among adolescents and are increasingly being recognized as a significant health problem in this age group [1,2]. Migraine and tension-type headache (TTH) are the most frequently reported types of primary headaches, with prevalence rates of approximately 10% for migraine and 15-20% for TTH in population-based studies [3-6]. Assessment of co-morbid disorders is important in order to improve the management of adolescents with recurrent headaches. Epidemiological studies in children and adolescents have shown that headache is associated with anxiety and depression, and also with attention and conduct difficulties [7-9]. It is still under debate whether the pain might be considered the cause or the consequence of psychological symptoms [9], and results from longitudinal studies among adults suggest that the association between depression and migraine may be bi-directional [10-12] with possibly shared genetic factors [13]. Whether depression as well as anxiety and other psychological symptoms are more specifically related to migraine than to TTH is, however, not clarified [8,9,14,15].

Studying psychological correlates of headache in the general population is important for many reasons. Firstly, depression is one of the leading causes of disability worldwide [16] and psychological symptoms in adolescents suffering from headache may impact the outcome of conventional headache treatments. Furthermore, adolescents with headache and psychological problems use more health services than individuals without [17]. Understanding and recognizing this comorbidity may therefore result in improvements in patient management. Secondly, current knowledge of comorbidity of headache is largely based on clinical samples with a high risk of selection bias and limited generalizability to headache sufferers in general. The objective of the present study was to assess relationships of anxiety and depression, conduct and attention difficulties with recurrent headache in in a large-scale epidemiological study of adolescents. The study also assessed possible similar associations for different headache types; migraine and TTH.

## Methods

### Young-HUNT 1

During a two-year period from August 1995 to June 1997, all students in lower secondary school (aged 12 to 15 years) and upper secondary school (aged 16 to 20 years) in Nord-Trøndelag county in Norway were invited to participate in the youth part of the Nord-Trøndelag Health Study, Young-HUNT.

A detailed description of the study has been published previously [18]. In short, a total of 8984 adolescents aged 12 to 20 years (response rate 90%) completed a comprehensive self-administered questionnaire during one school hour, with several health-related questions. In addition 6174 of them underwent a headache interview in connection with a clinical examination performed at the schools during school hours within a month after completing the questionnaire. The intention was to interview the whole population in connection with the clinical examination, but the interviews were delayed until February 1996, which explains the lower number of interviewed individuals. During the ‘missed’ period, both rural and industrial areas were studied, and there were no major differences in sex and age distribution between the total questionnaire-based study population and the questionnaire-based population that were also interviewed. Trained nurses performed the interviews, and the students were asked if they in the past 12 months had experienced recurring headaches that were not related to cold, fever or any other disease. Two typical headache symptom history descriptions, one for migraine and one for TTH were then read to those students who reported having had recurrent headache, and they were asked to classify their headache according to the descriptions.

The descriptions contained typical features for both migraine and TTH in accordance with the International Headache Society (IHS) criteria. The students were also given a third alternative (“non-classifiable headache”) in case neither of the two descriptions resembled their own symptoms. Headache frequency during the past year was recorded according to the following categories: less than 1 day per month (less than monthly), 1–3 days per month (monthly), 1–5 days per week (weekly), or more than 5 days per week (daily).

The “recognition-based” headache diagnoses obtained by the nurses have previously been validated against extensive semi-structured interviews by neurologists [6]. In short, the overall change-corrected agreement (kappa) between the nurse and the neurologist interviews was 0.76 [confidence interval (CI); 0.66-0.86], which is considered good [19]. For migraine, the positive and negative predictive values were 89 and 90%, respectively, and the change-corrected agreement (kappa) was 0.72 (CI; 0.58-0.87). For TTH, positive and negative predictive values were 83 and 91%, respectively, and the change-corrected agreement (kappa) was 0.74 (CI; 0.62-0.87). For non-classifiable headache, the positive and negative predictive values were 96 and 71%, respectively, and the change-corrected agreement (kappa) was 0.67 (CI; 0.46-0.88) [6].

Symptoms of anxiety and depression were measured by Symptom Check List (SCL-5), a five-item scale based on SCL-25, proven reliable in previous studies [20,21]. The items concerning anxiety symptoms in the questionnaire were: Been constantly scared and uneasy, felt tense and restless, worried too much about different matters. Depressive symptoms: felt hopeless when thinking of the future, felt down or sad. All questions had four alternative responses ranging from one: “not at all” to four: “extremely”. All five items were included in an anxiety/depressive variable as SCL-5 makes distinction between anxiety and depressive symptoms impossible. A mean score of the SCL-5 questions was computed, giving a scale ranging from one to four. In the present study the SCL-5 reached a Cronbach’s alpha of 0.79.

Variables concerning behavioral problems (attention- and conduct difficulties) were derived from the school adjustment part of the questionnaire, including 14 items, described in previous studies [22,23]. The adolescents were asked: “Do any of these situations happen to you at school, or have they happened before?” with four alternative responses from one: “never” to four: “very often”. Attention difficulties were estimated by the question how often “do you have problems concentrating in class” with a distribution from one to four. Conduct difficulties included how often the participants “quarrel with the teacher”, “skip school”, “get into fights” and “get scolded by the teacher”. A mean score ranging from one to four was computed. Cronbach’s alpha for conduct difficulties was 0.59.

### Ethics

Participation in the study was voluntary and based on written statements of consent from all participants. In addition, written consent from the parents was required for students below 16 years of age. The Regional Committee for Medical and Health Research Ethics and the Norwegian Data Inspectorate Board approved the study.

### Statistical analyses

The participants were stratified in two age groups (12–14 years and 15–17 years) and the associations of recurrent headache with symptoms of anxiety and depression as well as with behavioral problems were estimated using logistic regression. Recurrent headache versus no headache was used as dependent variable in the main analyses. In supplementary analyses migraine, TTH and non-classifiable headache compared to no headache were used as dependent variables. The independent variables were included by hierarchic regression. Symptoms of anxiety and depression was added in the first step, attention difficulties in the second step and conduct difficulties in the third step. All analyses were adjusted for sex and for family condition/single parenthood (“no” if living together with both parents, otherwise “yes”). The same kinds of analyses were done using headache frequency (<monthly, monthly, weekly or daily) compared to no headache as dependent variables. Data analyses were performed with the SPSS, version 20 (SPSS, Chicago, IL).

## Results

**Figure 1:**
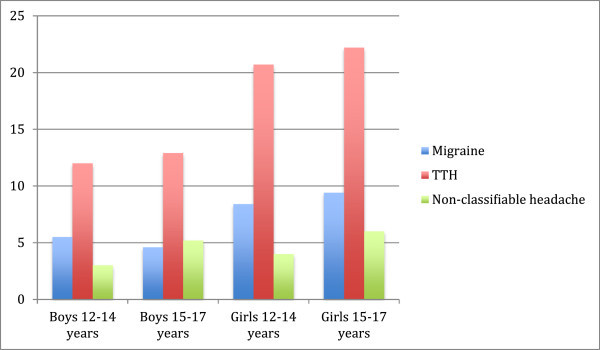
Prevalence (%) of headache diagnoses among boys and girls in different age groups.

Among the 6174 interviewed participants in Young-HUNT1, complete data on symptoms of anxiety and depression and behavioral problems were available for 5858 (94.9%), and we included the 4872 adolescents aged 12 to 17 years this study. Recurrent headache was reported by 1415 participants (29.0%), 902 girls (35.8%) and 513 boys (21.8%). The prevalence of headache diagnoses among boys and girls aged 12 to 14 years and 15 to 17 years is shown in Figure 1.

The overall mean score for symptoms of anxiety and depression was 1.35 (SD 0.43) among adolescents aged 12–14 years and 1.50 (SD 0.52) among adolescents aged 15–17 years. Attention difficulties had an overall mean score of 2.02 (SD 0.63) in the 12–14 years age group and of 2.26 (SD 0.66) in the 15–17 years age group. For conduct difficulties the mean score was 1.33 (SD 0.37) in the youngest age group and 1.37 (0.38) in the oldest group. In both age groups the scores for anxiety and depressive symtoms were higher among girls than boys (*p* < 0.001) and the scores for conduct difficulties were higher among boys than girls (*p* < 0.001), while there were no sex differences in the attention difficulties scores. In unadjusted analyzes stratified by age group and gender, adolescents with recurrent headache had higher scores for anxiety and depressive symptoms and behavioral difficulties than adolescents without headache (Table 1).

**Table 1:** Levels of anxiety and depressive symptoms, attention difficulties and conduct difficulties in girls and boys with and without recurrent headache

	**Boys (n = 2352)**				**Girls (n = 2520)**			
	**12-14 years (n = 952)**		**15-17 years (n = 1400)**		**12-14 years (n = 1002)**		**15-17 years (n = 1518)**	
	**Recurrent headache**	**Headache free**	**Recurrent headache**	**Headache free**	**Recurrent headache**	**Headache free**	**Recurrent headache**	**Headache free**
	**(n = 195)**	**(n = 757)**	**(n = 318)**	**(n = 1082)**	**(n = 331)**	**(n = 671)**	**(n = 571)**	**(n = 947)**
	**Mean (SD)**	**Mean (SD)**	**Mean (SD)**	**Mean (SD)**	**Mean (SD)**	**Mean (SD)**	**Mean (SD)**	**Mean (SD)**
**Symptoms of anxiety and depression**	1.39 (0.43)	1.25 (0.35)	1.50 (0.54)	1.33 (0.41)	1.53 (0.52)	1.37 (0.43)	1.73 (0.60)	1.55 (0.51)
**Attention difficulties**	2.08 (0.66)	1.94 (0.61)	2.42 (0.71)	2.20 (0,68)	2.12 (0.67)	2.03 (0.60)	2.35 (0,68)	2.20 (0.58)
**Conduct difficulties**	1.46 (0.43)	1.39 (0.40)	1.51 (0.46)	1.41 (0.40)	1.27 (0.34)	1.25 (0.29)	1.34 (0.35)	1.30 (0.32)

*Abbreviations:n* number; *SD* standard deviation.

In adjusted multivariate analyses among adolescents aged 12–14 years (Table 2) recurrent headache was significantly associated with symptoms of anxiety and depression, but not with behavioral problems. The same association with symptoms of anxiety and depression was found for all headache diagnoses (migraine, TTH and non-classifiable headache).

**Table 2:** Associations of recurrent headache and headache diagnoses with symptoms of anxiety and depression and behavioral problems among adolescents aged 12 to 14 years

	**Recurrent headache**		**Migraine**		**Tension-type headache**		**Non-classifiable headache**	
	**(yes = 526, no =1428))**		**(yes = 136, no = 1428)**		**(yes = 321, no = 1428)**		**(yes = 69, no = 1428)**	
	**OR (95% CI)**	***p*-value**	**OR (95% CI)**	***p*-value**	**OR (95% CI)**	***p*-value**	**OR (95% CI)**	***p*-value**
***Step 1***								
Symptoms of anxiety and depression	2.15 (1.71-2.70)	<0.001	1.69 (1.15-2.49)	0.007	2.20 (1.69-2.85)	<0.001	2.49 (1.58-3.91)	<0.001
***Step 2***								
Symptoms of anxiety and depression	2.05 (1.61-2.61)	<0.001	1.61 (1.07-2.42)	0.023	2.13 (1.62-2.82)	ns	2.22 (1.35-3.64)	0.002
Attention difficulties	1.11 (0.93-1.31)	ns	1.13 (0.84-1.51)	ns	1.07 (0.87-1.31)	ns	1.26 (0.86-1.86)	ns
***Step 3***								
Symptoms of anxiety and depression	2.05 (1.61-2.61)	<0.001	1.60 (1.06-2.41)	0.024	2.12 (1.61-2.80)	<0.001	2.24 (1.36-3.69)	0.002
Attention difficulties	1.10 (0.92-1.32)	ns	1.10 (0.80-1.51)	ns	1.05 (0.84-1.31)	ns	1.38 (0.91-2.09)	ns
Conduct difficulties	1.02 (0.75-1.40)	ns	1.11 (0.65-1.92)	ns	1.09 (0.75-1.59)	ns	0.64 (0.29-1.36)	ns

*Abbreviations:OR* odds ratio; *CI* confidence interval; *ns* non significant.

All analyses are adjusted for sex and single parenthood.

In the 15–17 years age group (Table 3), recurrent headache was significantly associated with symptoms of anxiety and depression and attention problems. Migraine was also associated with symptoms of anxiety and depression and attention problems, while TTH was associated with only anxiety and depressive symptoms. Non-classifiable headache was associated with both attention difficulties and behavioral difficulties, but not with symptoms of anxiety and depression.

**Table 3:** Associations of recurrent headache and headache diagnoses with symptoms of anxiety and depression and behavioral problems among adolescents aged 15 to 17 years

	**Recurrent headache**		**Migraine**		**Tension-type headache**		**Non-classifiable headache**	
	**(yes = 889, no =2029)**		**(yes = 207, no = 2029)**		**(yes = 518, no = 2029)**		**(yes = 164, no = 2029)**	
	**OR (95% CI)**	***p*-value**	**OR (95% CI)**	***p*-value**	**OR (95% CI)**	***p*-value**	**OR (95% CI)**	***p*-value**
***Step 1***								
Symptoms of anxiety and depression	1.84 (1.58-2.15)	<0.001	2.12 (1.65-2.72)	<0.001	1.77 (1.48-2.13)	<0.001	1.56 (1.16-2.10)	0.003
***Step 2***								
Symptoms of anxiety and depression	1.65 (1.40-1.93)	<0.001	1.78 (1.36-2.33)	<0.001	1.67 (1.37-2.04)	<0.001	1.30 (0.95-1.78)	ns
Attention difficulties	1.30 (1.14-1.48)	<0.001	1.51 (1.20-1.90)	<0.001	1.15 (0.98-1.35)	ns	1.53 (1.20-1.96)	0.001
***Step 3***								
Symptoms of anxiety and depression	1.64 (1.39-1.93)	<0.001	1.77 (1.35-2.32)	<0.001	1.67 (1.40-2.03)	<0.001	1.27 (0.92-1.74)	ns
Attention difficulties	1.25 (1.09-1.44)	0.001	1.47 (1.17-1.89)	0.001	1.12 (0.95-1.32)	ns	1.39 (1.07-1.81)	0.015
Conduct difficulties	1.21 (0.97-1.53)	ns	1.07 (0.72-1.62)	ns	1.14 (0.86-1.54)	ns	1.54 (1.03-2.3)	0.036

*Abbreviations:OR* odds ratio; *CI* confidence interval; *ns* non significant.

All analyses are adjusted for sex and single parenthood.

Among the 1415 adolescents aged 12–17 years reporting recurrent headache, data on headache frequency were available for 1350. Due to low numbers reporting daily headache, weekly and daily headache were combined. Table 4 shows the association between symptoms of anxiety and depression and behavioral problems in relation to headache frequency in the 12–14 years age group and Table 5 shows the same association in the 15–17 years group. There was a significant trend of an increasing symptom score and headache frequency, for both age groups with respect to anxiety and depressive symptoms (12–14 years, *p* < 0.001 and 15–17 years, *p* < 0.001) and attention difficulties (12–14 years, *p* < 0.001 and 15–17 years, *p* = 0.008), but not with conduct difficulties.

**Table 4:** Headache frequency in relation to levels of anxiety and depressive symptoms and behavioral problems among adolescents aged 12–14 years

	**Headache < monthly**		**Headache monthly**		**Headache weekly or daily**	
	**(yes = 97, no = 1428)**		**(yes = 269, no = 1428)**		**(yes = 133, no = 1428)**	
	**OR (95% CI)**	***p*-value**	**OR (95% CI)**	***p*-value**	**OR (95% CI)**	***p*-value**
***Step 1***						
Symptoms of anxiety and depression	1.37 (0.86-2.20)	ns	1.91 (1.43-2.55)	<0.001	3.39 (2.44-4.73)	<0.001
***Step 2***						
Symptoms of anxiety and depression	1.50 (0.91-2.46)	ns	1.82 (1.34-2.47)	<0.001	2.98 (2.08-4.26)	<0.001
Attention difficulties	0.84 (0.58-1.21)	ns	1.13 (0.90-1.41)	ns	1.33 (1.00-1.76)	0.049
***Step 3***						
Symptoms of anxiety and depression	1.50 (0.91-2.46)	ns	1.81 (1.33-2.46)	<0.001	2.99 (2.08-4.28)	<0.001
Attention difficulties	0.87 (0.59-1.30)	ns	1.10 (0.87-1.40)	ns	1.34 (0.99-1.81)	ns
Conduct difficulties	0.82 (0.42-1.61)	ns	1.10 (0.72-1.66)	ns	0.97 (0.58-1.65)	ns

*Abbreviations:CI* confidence interval; *OR* odds ratio; *ns* non significant.

The analyses were adjusted for sex and single parenthood.

**Table 5:** Headache frequency in relation to levels of anxiety and depressive symptoms and behavioral problems among adolescents aged 15–17 years

	**Headache < monthly**		**Headache monthly**		**Headache weekly or daily**	
	**(yes = 181, no = 2029)**		**(yes = 431, no = 2029)**		**(yes = 239, no = 2029)**	
	**OR (95% CI)**	***p*-value**	**OR (95% CI)**	***p*-value**	**OR (95% CI)**	***p*-value**
***Step 1***						
Symptoms of anxiety and depression	1.08 (0.79-1.50)	ns	1.94 (1.60-2.35)	<0.001	2.22 (1.75-2.80)	<0.001
***Step 2***						
Symptoms of anxiety and depression	1.03 (0.73-1.44)	ns	1.70 (1.38-2.09)	<0.001	1.91 (1.49-2.47)	<0.001
Attention difficulties	1.14 (0.89-1.46)	ns	1.36 (1.15-1.61)	<0.001	1.41 (1.13-1.76)	0.002
***Step 3***						
Symptoms of anxiety and depression	1.02 (0.73-1.43)	ns	1.68 (1.37-2.07)	<0.001	1.90 (1.47-2.45)	<0.001
Attention difficulties	1.09 (0.84-1.42)	ns	1.31 (1.10-1.57)	0.003	1.34 (1.06-1.70)	0.015
Conduct difficulties	1.22 (0.80-1.87)	ns	1.23 (0.92-1.65)	ns	1.27 (0.86-1.88)	ns

*Abbreviations:CI* confidence interval; *OR* odds ratio; *ns* non significant.

The analyses were adjusted for sex and single parenthood.

## Discussion

This is the first large-scale population-based study in Norway assessing adolescents’ recurrent headache in relation to symptoms of anxiety and depression and behavioral problems. Recurrent headache was associated with higher levels of symptoms of anxiety and depression in adolescents aged 12–14 years and 15–17 years evident for all headache diagnoses, except from non-classifiable headache among adolescents aged 15–17 years. Attention difficulties were associated with migraine and non-classifiable headache among those between 15–17 years of age. Conduct difficulties were only associated with non-classifiable headache in age group 15–17 years.

The results from the present population-based study confirm findings from clinic and community studies among children and adolescents indicating a relationship between psychological symptoms and recurring headache [8,9,24], but it is unclear whether this relationship is stronger for migraine than TTH [25,26]. A recent meta-analysis found more internalizing symptoms (mainly anxiety and depression) in patients with migraine and in patients with TTH than healthy controls, and no difference between headache types [27].

Regarding behavioral problems, results from earlier studies are more diverging. In a population-based cross-sectional study among adolescents aged 13–17 years, there was a significant association between headache and psychopathological symptoms [9]. Emotional symptoms and hyperactivity/inattention was associated with migraine and miscellaneous headache, but not with pure TTH. This is consistent with the results from the present study, as attention- and conduct problems were not associated with TTH. In previous clinical based studies, however, higher scores for both emotional and behavioral symptoms have been reported in children and adolescents with either migraine or TTH compared to healthy controls [7], more behavioral difficulties among those with TTH when compared to those with migraine [8] and higher scores for emotional symptoms and hyperactivity/inattention among adolescents with TTH when compared to headache-free controls [28]. A meta-analysis reports higher scores in externalizing symptoms (mainly behavioral problems) among migraine patients, but not for TTH when compared to healthy controls [27]. An important reason for variability in estimates of psychopathology in the pediatric and adolescent headache population is likely to be the differing diagnostic criteria used, both for the type of headache condition and the psychiatric symptoms.

Headache frequency was associated with increasing odds for symptoms of anxiety and depression, attention and conduct difficulties. This suggests that the degree of these symptoms may be more related to the frequency and severity of headache than to headache subtype. It is previously reported higher prevalence of comorbid psychiatric disorders in chronic daily headache than in other headache subtypes both in children/adolescents and adults [29,30]. Whether the association is specifically linked to recurrent headache, or to headache as a chronic pain is unclear.

Comorbidity of migraine and psychiatric disorders has been extensively studied, but the mechanisms underlying this phenomenon are far from clear. The possible mechanisms of comorbidity are several. Symptoms of anxiety and depression may develop as a result of migraine or vice versa, shared environmental risk factors may underlie both disorders and genetic or environmental risk factors may produce a brain state resulting in both conditions. The association of two disorders may also be a result of chance, which is less likely, since a meta-analysis of studies investigating the association of migraine and depression showed that depression was almost two times more frequent in subjects (aged 15–85 years) with migraine than in people unaffected by headache. Findings in this review support the view that migraine and psychiatric comorbidity is bi-directionally linked [25]. However, not all studies of children and adolescents confirm this comorbidity. In a recent systematic review, there was no evidence that children and adolescents with migraine at referral to a specialist had more signs of anxiety and depression, had more attention problems or exhibited more externalizing behavior than healthy children [31].

The strengths of the study include the large and unselected population, the relatively high participation rate, and the use of validated headache diagnoses. There was no systematic selection of the interviewed participants, which indicates that the study cohort represents the population fairly well. The study also has some inherent limitations. It is not always easy to distinguish between migraine and TTH, especially not in children and adolescents. Altogether it has been debated whether such distinctions need to be made because there seem to be more similarities than differences [26]. Although we used a validated “recognition-based” headache diagnostic method in our study, misclassification may have had an impact on the supplementary analyses. The questionnaires that were used to measure symptoms of anxiety and depression and behavioral problems have well known advantages as well as limitations [21]. A questionnaire method does not produce any clinical diagnosis, which is one of the important limitations of the present study.

## Conclusion

In conclusion, this population-based study indicates that symptoms of anxiety and depression and behavioral problems are associated with recurrent headache among Norwegian adolescents. An increased awareness among clinicians might lead to better headache treatment and management of adolescents with recurrent headaches.

## Competing interests

The authors declare that they have no competing interests.

## Authors’ contributions

J-AZ and GD conceived of the study, and participated in its design and coordination and helped to draft the manuscript. TW-L supervised the statistical analyses. TLH, KH, and ML participated in the design of the study and revised the manuscript critically. All authors read and approved the final manuscript.
